# Solid Pseudopapillary Neoplasm of the Pancreatic Head With Atypical Presentation in a Young Woman: A Case Report

**DOI:** 10.7759/cureus.108133

**Published:** 2026-05-02

**Authors:** Jaysoom W Abarca Ruiz, Cintya A Borja Velasco, Mauricio N Suárez Caicedo, Estefanía A Arteaga Morocho, Edgar J Redroban Tufino, Janneth Aracely Valdivieso Maggi, Patricia C Corella Sanguil, Ismael P Viteri Paredes, Leslie A Barrera Idrovo, Lincey G Salazar Quevedo

**Affiliations:** 1 Gastroenterology, Hospital de Especialidades Eugenio Espejo, Quito, ECU; 2 Gastroenterology, Hospital Metropolitano, Quito, ECU; 3 Gastroenterology, Pontificia Universidad Católica del Ecuador, Quito, ECU; 4 Gastroenterology, Universidad de las Américas, Quito, ECU

**Keywords:** computed tomography, immunohistochemistry, incidental findings, pancreatic neoplasms, pancreaticoduodenectomy, pseudopapillary pancreatic neoplasms, solid pseudopapillary neoplasm of the pancreas

## Abstract

Solid pseudopapillary neoplasm of the pancreas is a rare low-grade malignant tumor that predominantly affects young women and often presents with nonspecific or absent symptoms, which may delay diagnosis. We report the case of a 22-year-old female patient with no relevant medical history who was referred after the incidental finding of a pancreatic mass on imaging studies. Initially asymptomatic, the lesion progressed during follow-up to a painful and palpable abdominal mass. Computed tomography and endoscopic ultrasound revealed an 8-cm heterogeneous lesion located in the head of the pancreas. Given its size, location, and progressive growth, the patient underwent surgical resection, including proximal subtotal pancreatectomy with total duodenectomy, partial antrectomy, and pancreatoduodenectomy. Intraoperative findings revealed a 15 × 12 cm encapsulated tumor with reactive lymphadenopathy. Histopathological examination confirmed a grade G1 solid pseudopapillary neoplasm staged as pT4 pN0 pMx. Postoperatively, the patient developed exocrine pancreatic insufficiency requiring enzyme replacement therapy and remains under regular oncological follow-up without evidence of recurrence. This case highlights the progressive growth of an initially asymptomatic lesion and underscores the importance of early recognition and surgical management. The atypical location in the pancreatic head further emphasizes the need to consider this diagnosis even in unusual anatomical presentations.

## Introduction

Solid pseudopapillary neoplasm of the pancreas (SPN), also known as Frantz tumor, is a rare epithelial neoplasm first described in 1959 and currently classified by the World Health Organization as a low-grade malignant tumor due to its potential for local invasion and metastasis [1,2].

Although it represents a small proportion of pancreatic neoplasms, its clinical importance lies in its favorable prognosis after complete surgical resection, with long-term survival rates exceeding 90% [3,4]. This contrasts with other pancreatic tumors, which are typically associated with poor outcomes.

The pathogenesis of SPN is not fully understood; however, mutations in the CTNNB1 gene leading to abnormal β-catenin accumulation are a key feature. This alteration is clinically relevant, as it supports the diagnosis through characteristic nuclear β-catenin expression on immunohistochemistry [1,5].

Despite advances in imaging techniques, SPN remains a diagnostic challenge due to its variable presentation and overlap with other pancreatic lesions, particularly within the spectrum of cystic pancreatic neoplasms and neuroendocrine tumors [6,7]. Atypical features such as unusual tumor location, large size, or progressive clinical evolution may further complicate early recognition and therapeutic decision-making.

In this context, we present the case of a young adult patient with an incidentally detected pancreatic mass that demonstrated progressive growth and atypical localization in the pancreatic head. This case highlights an uncommon clinical and anatomical presentation that may complicate early recognition and influence surgical decision-making.

## Case presentation

A 22-year-old female university student with no relevant past medical or surgical history and a gynecological history of one prior pregnancy and use of a subdermal contraceptive implant for approximately one year, which may be of interest given the known progesterone receptor expression in solid pseudopapillary neoplasms, was referred from a primary care center after the incidental finding of a pancreatic mass on a non-contrast abdominal computed tomography (CT) scan performed in July 2024 during a general evaluation. At the time of diagnosis, the patient was asymptomatic, denying abdominal pain, weight loss, or constitutional symptoms.

Initial physical examination revealed a soft, depressible, and non-tender abdomen, without palpable masses. Baseline laboratory evaluation showed no significant abnormalities as a complete blood count demonstrated normal values of leukocytes, hemoglobin, hematocrit and platelet count. Renal function and serum glucose were within normal limits (Table 1).

**Table 1:** Laboratory findings AST: Aspartate Aminotransferase; ALT: Alanine Aminotransferase; CA: Cancer Antigen; CEA: Carcinoembryonic Antigen; AFP: Alpha-Fetoprotein

Parameter	Initial	Follow-up	Reference range
Leukocytes (/µL)	9.55 ×10³	15.05 ×10³	4–10 ×10³
Hemoglobin (g/dL)	15.3	15.5	12–16
Hematocrit (%)	46.9	47.3	36–46
Platelets (×10³/µL)	370	393	150–400
Urea (mg/dL)	43.4	30.3	10–50
Creatinine (mg/dL)	0.66	0.63	0.6–1.2
Glucose (mg/dL)	94.9	104	70–100
Total bilirubin (mg/dL)	—	0.46	0.2–1.2
AST (U/L)	—	23.5	<35
ALT (U/L)	—	27.3	<35
Albumin (g/dL)	—	4.73	3.5–5.0
Sodium (mmol/L)	—	140.7	135–145
Potassium (mmol/L)	—	4.26	3.5–5.0
Chloride (mmol/L)	—	104	98–107
CA 19-9 (U/mL)	—	7.10	<37
CEA (ng/mL)	—	0.59	<5
AFP (ng/mL)	—	1.44	<10
CA 15-3 (U/mL)	—	7.98	<30

**Figure 1:**
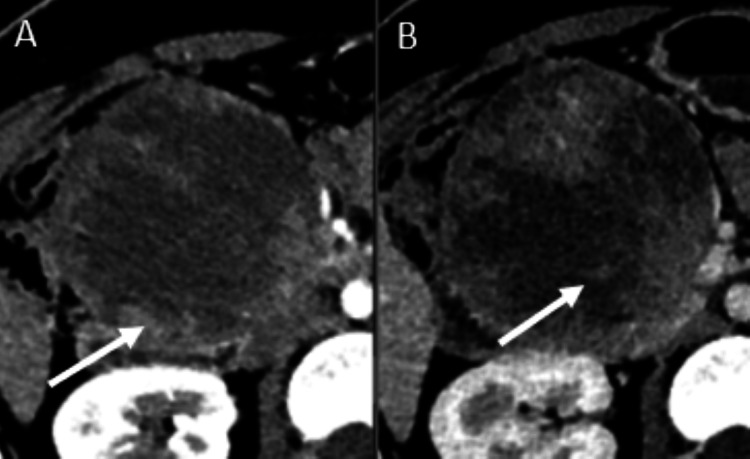
Contrast-enhanced CT demonstrating a pancreatic solid pseudopapillary neoplasm (A) Axial CT image showing a well-defined mass with peripheral enhancement (arrow) suggestive of a peripheral enhancing capsule; (B) The lesion demonstrates central hypodense areas (arrow) consistent with necrosis or cystic degeneration.

Contrast-enhanced abdominal CT revealed a well-defined, heterogeneous mass located in the head of the pancreas measuring approximately 80 × 79 mm, with peripheral calcifications and no evidence of vascular invasion or distant metastasis. The lesion displaced adjacent structures, including the duodenum, without evidence of biliary obstruction. These findings were suggestive of a solid pseudopapillary neoplasm (Figure 1), which was subsequently confirmed by histopathological and immunohistochemical evaluation.

Endoscopic ultrasound revealed a heterogeneous hypoechoic lesion with predominantly solid components (approximately 80%) and internal cystic areas in the uncinate process and pancreatic head, consistent with a solid pseudopapillary neoplasm.

During follow-up, two months after the initial evaluation, the patient developed a progressively enlarging abdominal mass that became clinically palpable, characterized as firm, non-mobile, and non-tender located in the epigastrium and right upper quadrant, associated with intermittent, non-radiating abdominal discomfort without clear relation to meals or body position. Repeat laboratory evaluation demonstrated leukocytosis with neutrophilia (this finding was interpreted as a possible inflammatory response related to tumor necrosis), while hemoglobin, hematocrit and platelet count remained within normal limits. Liver function tests were obtained during follow-up as part of a comprehensive evaluation after the development of symptoms; renal function and electrolytes were also within normal ranges. Tumor markers, including CA 19-9, carcinoembryonic antigen, alpha-fetoprotein and CA 15-3 (included as part of a broad tumor marker panel) were within normal limits (Table 1).

Given the size, location, and progressive growth of the lesion, which became clinically palpable and raised suspicion for a solid pseudopapillary neoplasm, the patient underwent exploratory laparotomy with adhesiolysis, subtotal proximal pancreatectomy with total duodenectomy, partial antrectomy, choledochoenterostomy, gastrojejunostomy, and pancreatojejunostomy (Whipple procedure). Intraoperative findings revealed an oval, encapsulated, calcified tumor measuring approximately 15 × 12 cm, completely involving the pancreatic head, with reactive lymphadenopathy and no evidence of major vascular invasion. Adhesions between the tumor and the transverse colon were observed.

**Figure 2:**
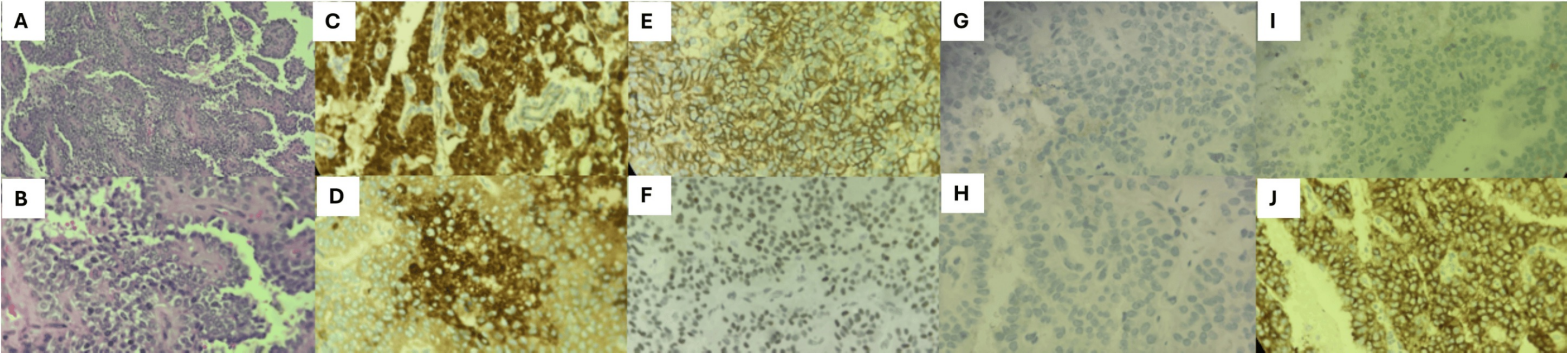
Histopathological and immunohistochemical features of the tumor (A) Hematoxylin and eosin (H&E), 10× magnification, showing a neoplasm composed of poorly cohesive cells arranged in pseudopapillary structures with delicate fibrovascular cores; (B) Hematoxylin and eosin (H&E), 40× magnification, demonstrating relatively uniform tumor cells with eosinophilic cytoplasm and round to oval nuclei, consistent with a well-differentiated neoplasm; (C) Immunohistochemistry for β-catenin showing strong nuclear and cytoplasmic positivity, characteristic of activation of the Wnt signaling pathway in solid pseudopapillary neoplasms; (D) Immunohistochemistry for alpha-1-antitrypsin demonstrating diffuse cytoplasmic positivity; (E) Immunohistochemistry for CD56 showing membranous positivity; (F) Immunohistochemistry for progesterone receptor showing nuclear positivity; (G) Immunohistochemistry for chromogranin showing negative staining, arguing against a neuroendocrine tumor; (H) Immunohistochemistry for cytokeratin 7 (CK7) showing negative staining;  (I) Immunohistochemistry for synaptophysin showing negative staining; (J) Immunohistochemistry for CD10 demonstrating membranous positivity.

Histopathological examination confirmed a solid pseudopapillary neoplasm, grade G1 (well differentiated), measuring 7 × 7.5 × 8 cm, confined to the pancreas, without lymphovascular or perineural invasion. All six resected lymph nodes were negative for malignancy, and the tumor was staged as pT4 pN0 pMx. The immunohistochemical profile supported the diagnosis, with nuclear positivity for β-catenin, reflecting activation of the Wnt signaling pathway, along with expression of alpha-1-antitrypsin, cluster of differentiation (CD)56, progesterone receptor, and CD10, consistent with the characteristic phenotype of this neoplasm (Figure 2).

The absence of chromogranin, synaptophysin, and cytokeratin 7 (CK7) expression effectively excluded a neuroendocrine tumor within the differential diagnosis (Table 2).

**Table 2:** Immunohistochemical profile CD: Cluster of Differentiation; CK: Cytokeratin.

Marker	Result
β-catenin	Positive (nuclear)
Alpha-1-antitrypsin	Positive
CD56	Positive
Progesterone receptor	Positive (nuclear)
CD10	Positive
Chromogranin	Negative
Synaptophysin	Negative
CK7	Negative

In the immediate postoperative period, the patient developed hypovolemic shock requiring fluid resuscitation and vasopressor support, with subsequent stabilization and admission to the intensive care unit. She showed favorable clinical evolution and was discharged seven days after surgery.

During follow-up, the patient reported progressive improvement in abdominal symptoms, particularly in relation to pain and functional limitation. However, she developed exocrine pancreatic insufficiency, manifested by steatorrhea, bloating, and postprandial abdominal discomfort, likely secondary to the extent of pancreatic resection. The diagnosis was established clinically based on symptoms, as no quantitative testing was performed. Pancreatic enzyme replacement therapy was initiated with a favorable clinical response, resulting in improved stool consistency, reduction of gastrointestinal symptoms, and better nutritional tolerance.

Nutritional counseling was also provided, with emphasis on adequate caloric intake and fat-adjusted diet, contributing to stabilization of her clinical condition. The patient has remained clinically stable, with no evidence of endocrine pancreatic insufficiency or need for insulin therapy to date.

**Figure 3:**
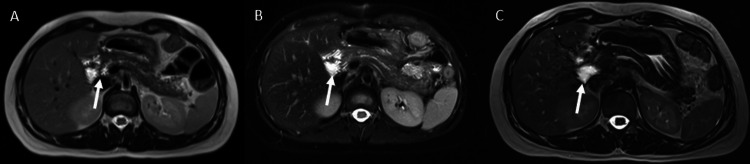
Postoperative magnetic resonance imaging demonstrating changes after pancreatoduodenectomy (A–C) Axial magnetic resonance images obtained during postoperative follow-up showing expected postsurgical changes in the pancreatic head region following pancreatoduodenectomy. Areas of altered signal intensity (arrows) correspond to postoperative changes, including fibrosis and surgical reconstruction. No discrete mass lesion is identified, and there is no radiological evidence of tumor recurrence or metastatic disease.

She continues under regular outpatient follow-up by gastroenterology and oncology services, with periodic clinical and imaging surveillance. There is no evidence of disease recurrence according to the most recent magnetic resonance imaging performed six months after surgery (Figure 3).

Her overall functional status remains preserved, allowing her to return to daily activities and academic routine.

## Discussion

SPN is a rare pancreatic tumor that accounts for approximately 0.9-2.7% of all exocrine pancreatic neoplasms and up to 5% of pancreatic cystic neoplasms, with a mean age at presentation of approximately 28.5 years and a marked predominance in young women, with a female-to-male ratio of 9.8:1 [1,2].

One of the most relevant aspects of the present case was its clinical evolution. Unlike what is described in the literature, where these neoplasms usually remain asymptomatic and are diagnosed incidentally, in our patient the tumor was initially identified as an incidental imaging finding without associated symptoms. During follow-up, progression of the mass effect was observed until it became clinically palpable and painful, this relatively rapid progression over a short period contrasts with the typically indolent course described in the literature and highlights the variability in clinical behavior of solid pseudopapillary neoplasms. This transition from a subclinical phase to structural manifestations detectable on physical examination represents an uncommon evolution and suggests slow but progressive tumor growth until reaching a size that allowed clinical detection [1].

From an anatomical standpoint, tumor location in the pancreatic head is also atypical in relation to the patient’s age. In young adult women, SPN are predominantly described in the pancreatic body and tail, whereas cephalic location is more common in pediatric or adolescent populations [1]. In this sense, the presence of a large lesion in the pancreatic head in a young adult represents an epidemiological discordance that may have contributed both to symptom development and the need for a more extensive surgical approach.

Regarding imaging evaluation, CT usually shows these lesions as heterogeneous structures with peripheral solid components and central cystic areas, which may be accompanied by calcifications [6,8]. In the present case, although the tomographic findings were consistent with the literature, a notable discrepancy was observed between the size estimated by imaging (8 cm) and the intraoperative finding (up to 15 cm), suggesting possible radiological underestimation in encapsulated tumors. This phenomenon has been described in tumors with mixed solid-cystic architecture where imaging may not fully reflect the true tumor extent [8].

Definitive diagnosis relies on histopathological examination. In this case, the characteristic pattern of monomorphic cells arranged in pseudopapillary structures with fibrovascular cores and pseudorosettes was observed, considered highly suggestive of this entity [1,4]. The immunohistochemical profile further supported the diagnosis, demonstrating nuclear positivity for β-catenin, consistent with activation of the Wnt signaling pathway, along with expression of alpha-1-antitrypsin, CD56, progesterone receptor, and CD10. The absence of chromogranin and synaptophysin expression effectively excluded a neuroendocrine tumor within the differential diagnosis [1]. Although identification of CTNNB1 gene mutations may be sufficient for diagnosis, its availability remains limited in many centers [1]. It is important to note that the pathological staging in this case may not strictly correlate with conventional Tumor, Node, and Metastasis (TNM) criteria used for pancreatic ductal adenocarcinoma. Solid pseudopapillary neoplasms may be staged differently in pathological reports, and classification can vary depending on institutional or interpretative criteria. In this case, although the tumor was confined to the pancreas and showed no evidence of vascular invasion, it presented adhesions to the transverse colon, which contributed to its classification as pT4 in the original report.

From a therapeutic standpoint, standard treatment is complete surgical resection, preserving pancreatic parenchyma when possible depending on tumor location [1,5]. However, in tumors located in the pancreatic head, especially when large or involving adjacent structures, more radical procedures such as pancreatoduodenectomy may be required. In this case, the combination of cephalic location and significant tumor volume led to the choice of a Whipple resection, allowing complete removal with negative margins, which remains the main determinant of favorable prognosis in these neoplasms [5].

Prognosis is generally excellent after complete resection, with five- and 10-year survival rates of 96% and 93%, respectively. Nevertheless, between 10% and 15% of cases may exhibit malignant behavior or metastasize. Although tumor size, lymphovascular invasion, and Ki-67 index have been proposed as predictors of aggressiveness, evidence remains limited [1,5]. In this context, recognition of atypical clinical presentations, such as that observed in this case, may influence clinical suspicion and management decisions. Additionally, this relatively rapid progression over a short period contrasts with the typically indolent course described in the literature and highlights the variability in clinical behavior of solid pseudopapillary neoplasms. Longer follow-up is required to assess long-term outcomes and recurrence risk.

This case has some limitations. The follow-up period is relatively short, which precludes assessment of long-term outcomes and potential late recurrence. Additionally, the absence of quantitative assessment of exocrine pancreatic insufficiency limits objective evaluation of postoperative functional impact. The potential influence of hormonal exposure, including the use of a subdermal contraceptive implant, could not be established, although it may be of interest given the known expression of progesterone receptors in this type of tumor. Furthermore, as a single case report, the findings may not be generalizable but are intended to contribute to the recognition of atypical clinical presentations.

## Conclusions

SPN is a rare entity that typically follows an indolent course and is often diagnosed incidentally, particularly in young women. This case highlights an uncommon clinical evolution in which an initially asymptomatic lesion progressed to a large, palpable mass over a short period, reflecting variable tumor behavior.

The atypical localization in the pancreatic head and the significant tumor size influenced the need for extensive surgical management. Despite these features, the absence of biochemical abnormalities and normal tumor markers reinforces the importance of imaging in the evaluation of pancreatic masses.

The patient demonstrated a favorable short-term outcome, with no evidence of recurrence at six months, consistent with the known good prognosis after complete surgical resection. Careful follow-up with periodic imaging, such as annual studies, may be considered to monitor for potential recurrence.
